# Factors attributed to the higher in-hospital mortality of ST elevation myocardial infarction patients admitted during off-hour in comparison with those during regular hour

**DOI:** 10.1371/journal.pone.0175485

**Published:** 2017-04-07

**Authors:** Min Li, Shenshen Li, Xin Du, Tao Wu, Xian Li, Changsheng Ma, Yong Huo, Dayi Hu, Runlin Gao, Yangfeng Wu

**Affiliations:** 1 Department of Epidemiology and Biostatistics, School of Public Health, Peking University, Beijing, China; 2 The George Institute for Global Health at Peking University Health Science Center, Beijing, China; 3 Beijing Anzhen Hospital, Capital Medical University, Beijing, China; 4 Peking University First Hospital, Beijing, China; 5 Peking University People’s Hospital, Beijing, China; 6 The Department of Cardiology, Cardiovascular Institute and Fuwai Hospital, Chinese Academy of Medical Sciences and Peking Union Medical College, Bejing, China; University of Louisville, UNITED STATES

## Abstract

**Background:**

In-hospital mortality of patients with ST elevation myocardial infarction (STEMI) admitted during off-hour was reported higher than those admitted during regular hour, but which factors cause the difference remains largely unknown though the difference in medical resources was often accused.

**Methods and results:**

This registry-based study recruited 7456 STEMI patients prospectively from 99 level two hospitals across China. Generalized linear mixed models were applied to quantify the risk of in-hospital death attributed to admission time and the explainers of its change, accounting for the clustering of patients within hospitals. There were 45.2% patients admitted during regular hour and 54.8% during off-hour. In-hospital mortality was 7.0% for patients admitted during regular hour and 8.3% for those during off-hour (p<0.05). Generalized linear mixed models adjusting for age, gender and education showed that patients’ disease severity at admission and medical treatments received after admission could explain the risk difference attributed to admission time by 55% and 20%, respectively. After all factors accounted, the residual relative risk difference left only 6% (adjusted OR = 0.94) and became no longer significant.

**Conclusions:**

The regular-and-off-hour mortality difference exists among STEMI patients in Chinese level two hospitals, which could be attributed primarily to disease severity at admission and secondly to the poorer medical treatments. These results call for public attention to the more severity of STEMI patients admitted during off-hour in addition to improving medical resources for STEMI at off-hour.

## Introduction

Patients with ST elevation myocardial infarction (STEMI) admitted during off-hour have increased mortality in comparison with their counterparts admitted during regular hour. Such ‘weekend effect’ was first recognized in Rogot et al’s study and confirmed by other studies mostly in North America and Europe [1–2]. Considering the widespread promulgation and endorsement of STEMI treatment guidelines, the ‘weekend effect’ for STEMI might diminish over time. A recent report on data from Healthcare Cost and Utilization Project Nationwide Inpatient Sample showed that the ‘weekend effect’ among acute myocardial infarction patients in United States has gone due to greater adherence to the benchmarks for timelines of diagnosis and therapy [3]. But the situation in other countries, particularly in settings where medical resources are limited remains unknown. These regions often experience a higher mortality of STEMI [4]. In China, about 45% of population resides in rural region, with access only to resource-constrained hospitals, and there were reports that in-hospital mortality for patients with acute coronary syndrome in level two hospitals was higher than the patients in tertiary hospitals [5–6].

To understand reasons that caused the ‘weekend effect’, previous studies mostly focused on the problems in health system and quality of healthcare. It is widely accepted that the excess risk of death during off-hour admission may be attributed to a lower rate of invasive cardiac procedures and longer door to balloon time due to reduced staff resources, both number of staff and their expertise on site [7–10]. However, it is still largely unknown whether or to what extent the difference in patients’ clinical features at admission such as disease severity and the history and risk factors of cardiovascular disease could account for the higher mortality of off-hour admission.

Herein, the present study is to confirm whether the ‘weekend effect’ exists among Chinese patients with STEMI, and to understand the factors, if there are, that result in this mortality difference. The information gained from the study could guide the policy making and clinical guidelines development that finally help to eliminate the excess death in patients admitted during off-hour.

## Materials and methods

### Data source and study population

We used data from the Clinical Pathways in Acute Coronary Syndromes—phase 3 (CPACS-3) study. The study protocol has been published previously [11]. Briefly, CPACS-3 was a stepped-wedge cluster-randomized trial that sought to provide rigorous evidence to inform the quality of care improvement initiative in the management of acute coronary syndrome. The study recruited 104 remote level two hospitals (broadly defined as regional hospitals providing medical services to several communities) without percutaneous coronary intervention facilities from 15 provinces throughout China. Ninety-nine hospitals were finally included for 2 refused to participate and 3 were removed for not meeting the quality requirements. Patients aged ≥18 years with a final diagnosis of acute coronary syndrome identified at the time of death or discharge were prospectively registered between December 2011 and December 2014. Patients were excluded if they were dead on arrival or died within 10 minutes after arriving at hospital. The medical charts of eligible patients were reviewed to collect requested information by centrally trained and certified research personnel who were not involved in the clinical care of the patients, and then entered into a web-based CPACS-3 Data Management System.

For this paper, we restricted the analysis to STEMI patients with complete initial assessment at admission. Of the 7728 eligible patients, 13 patients were excluded for missing accurate information of arrival date or arrival time, and 259 were excluded for being transferred to another hospital within 24 hours of admission. The remaining 7456 patients were included in the present study.

### Study variables

The information collected on each patient included demographic characteristic, disease history, cardiovascular risk factors, hours from onset to admission, disease severity at admission, thrombolytic procedure, medications therapy, complications and discharge outcome. Details of these variables are shown in Table 1.

**Table 1:** Characteristics of patients with STEMI admitted during regular and off-hour.

Variables	Regular hour (n = 3370)	Off-hour (n = 4086)	P value
Demographic characteristics			
Age, mean (SD)	64.0 (12.3)	63.3 (12.5)	0.02
Male, %	69.5	71.7	0.03
Education (middle school or higher), %	31.9	31.9	0.99
History of disease			
Myocardial infarction, %	4.9	5.9	0.06
Angina, %	11.2	11.0	0.78
Stroke, %	9.0	9.1	0.86
Heart failure, %	2.1	2.7	0.09
Hypertension, %	41.3	41.7	0.71
Dyslipidemia, %	4.1	3.6	0.29
Diabetes, %	11.3	11.6	0.70
Chronic kidney disease, %	0.7	0.9	0.39
Risk factors			
Current smoker, %	34.1	35.3	0.25
Obesity, %	6.6	7.8	0.12
Systolic blood pressure (mmHg), mean (SD)	129.2 (28.0)	130.5 (29.2)	0.05
Diastolic blood pressure (mmHg), mean (SD)	80.9 (18.0)	81.8 (18.4)	0.04
Total cholesterol (mmol/L), mean (SD)	4.6 (1.2)	4.6 (1.2)	0.15
Low density lipoprotein cholesterol (mmol/L), mean (SD)	2.7 (1.0)	2.8 (1.0)	0.04
High density lipoprotein cholesterol (mmol/L), mean (SD)	1.2 (0.5)	1.2 (0.4)	0.46
Triglycerides (mmol/L), mean (SD)	1.6 (1.3)	1.6 (1.3)	0.41
Hours from onset to admission, hour ^a^	5.3 (2.0–27.7)	3.3 (1.5–12.0)	< .01
Arrival within 12 hours, %	62.7	75.1	< .01
Clinical features at admission			
Systolic blood pressure <90mmHg, %	4.4	5.4	0.05
Heart rate> = 100beats/min, %	11.3	12.9	0.04
Killip, %			0.09
I	65.2	64.2	
II	19.6	18.9	
III	8.1	8.2	
IV	7.2	8.7	
Cardiopulmonary resuscitation within 24h, %	4.1	5.1	0.04
Abnormal rhythm of heart within 24h,%	9.7	12.4	< .01
Thrombolytic therapy			
% receiving the therapy	34.8	43.1	< .01
% with door-to-needle < = 30min	33.8	30.8	0.09
% success of thrombolytic therapy	83.5	82.8	0.89
Medications within 24 hours			
Aspirin, %	95.6	94.0	< .01
Clopidogrel, %	88.0	86.7	0.09
ACEI/ARB, %	49.4	46.6	0.02
Beta-blocker, %	54.2	50.4	< .01
Statin, %	92.0	90.7	0.04
Nitrates, %	76.7	75.8	0.40
Calcium antagonist, %	4.3	4.7	0.40
Heparin/low density lipoprotein heparin, %	88.3	89.1	0.23
Complications			
Heart failure, %	10.2	10.7	0.50
Arrhythmia, %	8.7	9.8	0.10
Bleeding, %	0.7	0.9	0.49
Reoccur myocardial infarction, %	0.7	0.5	0.27
Stroke, %	0.1	0.1	0.78
Outcomes			
In-hospital death, %	7.0	8.3	0.04
Transferring to tertiary hospitals, %	17.5	17.6	0.85

^a^ presented as medians and inter quartile ranges (25-75th).

ACEI: angiotensin converting enzyme inhibitors; ARB: angiotensin receptor blocker; STEMI: ST elevation myocardial infarction

These patients were classified into two groups: regular hour group where admission time were between 08:00am and 17:00pm during weekdays (Monday to Friday); off-hour group where patients were admitted during weekday between 17:01pm and 07:59am, and during any time on weekends (Saturday and Sunday) and Chinese statutory holidays.

The primary outcome was in-hospital all-cause mortality. Variables indicating disease severity at admission included hypotension (systolic blood pressure<90mmHg), tachycardia (heart rate> = 100beats/min), Killip class, cardiopulmonary resuscitation within 24 hours of admission, and abnormal rhythm of heart within 24 hours of admission (ventricular tachycardia, ventricular fibrillation, or atrial fibrillation is detected from continuous electrocardiogram monitoring). The detail definitions of main variables are summarized in S1 Table.

### Statistical analyses

Comparisons of clinical characteristics between regular hour and off-hour group were made by using chi-square test for categorical variables and Student’s t-test or Wilcoxon rank sum test for continuous variables, as appropriate.

In examining the relationship between admission time and in-hospital mortality, we adjusted for variables that were significantly different between regular hour and off-hour group as in Table 1 and variables of known prognostic importance. These included demographic characteristics, disease history, risk factors, hours from onset to admission, disease severity at admission, and medical treatments. Generalized linear mixed model was used to fit the analysis model, with a random effect of hospitals to account for the correlation of patients within hospitals. Those variables with a P value greater than 0.05 would be stepped out from the optimal model.

To further address the contribution of each covariate that could account for the mortality difference of admission time, we first fitted the base risk model of in-hospital death with demographic characteristics (age, gender and education) included in addition to the admission time. We used 1-OR of admission time from the base model as the relative risk difference attributed to admission, since the in-hospital mortality could be considered as an event with low frequency. We then added each potential explaining variable set including disease history and risk factors, disease severity, medical treatments, and all these variables together into the base model, and observed the change in relative risk difference attributed to admission time.

The change in relative risk difference attributed to admission time was calculated using the following formula:

$$\frac{\left|\left(1\text{-}{\text{OR}}_{\text{i}}\right)-\left(1\text{-}{\text{OR}}_{\text{b}}\right)\right|}{1\text{-}{\text{OR}}_{\text{b}}}\times 100\%$$

Where OR_b_ represents OR of admission time from base mode and OR_i_ represents OR of admission time after adding explaining variables [12].

For all analyses, P <0.05 were considered to be significant. All analyses were done using SAS version 9.4 (SAS Inc., Cary, NC, USA).

### Ethics approval

Ethics approval for CPACS-3 was obtained from Peking University Institutional Review Board. All participants provided written informed consents. Patients’ data in the Data Management System are protected by password and only de-identified data are available to users designated by the study with appropriate authorization levels.

## Results

### Clinical characteristics of study population

Among 7456 STEMI patients, 3370 (45.2%) patients admitted during regular hour and 4086 (54.8%) admitted during off-hour. The study patients were predominantly male (71%) and the mean age was 63.5 years.

Table 1 presented the demographics and clinical characteristics of study patients by admission time. Patients in regular hour group were similar with those in off-hour group in terms of education, disease history, risk factors including current smoker, obesity and lipids level, as well as complications. However, patients in off-hour group were slightly younger, more likely to be male, had shorter hours from onset to admission but significantly more likely to have hypotension, tachycardia, Killip IV, cardiopulmonary resuscitation and abnormal rhythm of heart within 24 hours of admission. They also received more thrombolytic therapy but fewer medications including aspirin, angiotensin converting enzyme inhibitors or angiotensin receptor blocker, beta-blocker, and statin within 24 hours of admission.

### In-hospital mortality

Patients in regular hour group had significantly lower mortality than those in off-hour group (Table 1). The unadjusted OR of in-hospital mortality was 0.82 (95%CI: 0.69–0.98) for regular hour admission in comparison with off-hour admission. In a generalized linear mixed model with all variables adjusted, the associations of in-hospital mortality with admission time became non-significant (P>0.05). The significant predictors of in-hospital all-cause mortality remained in the model are showed in Table 2.

**Table 2:** Predictors of in-hospital mortality for STEMI patients in generalized linear mixed model.

Variables	Adjusted OR (95%CI)
Age	1.05 (1.04–1.06)
Gender (male vs female)	0.65 (0.53–0.81)
Education (middle school or higher vs lower than middle school)	0.59 (0.45–0.79)
Disease history and risk factors	
History of stroke	1.63 (1.22–2.18)
Obesity (yes vs no)	1.53 (0.86–2.73)
Obesity (unmeasured vs no)	1.55 (1.20–1.99)
Severity at admission	
Systolic blood pressure <90mmHg	1.54 (1.07–2.21)
Heart rate> = 100beats/min	1.59 (1.24–2.04)
Killip (II vs I)	0.96 (0.72–1.28)
Killip (III vs I)	2.12 (1.55–2.90)
Killip (IV vs I)	2.97 (2.21–3.99)
Cardiopulmonary resuscitation within 24h	13.64 (10.00–18.60)
Abnormal rhythm of heart within 24h	2.09 (1.61–2.70)
Treatment	
Thrombolytic (door-to-needle<30min vs no thrombolytic)	0.48 (0.31–0.74)
Thrombolytic (door-to-needle>30min vs no thrombolytic)	0.99 (0.76–1.28)
Aspirin within 24h	0.48 (0.34–0.67)
ACEI/ARB within 24h	0.79 (0.63–0.98)

ACEI: angiotensin converting enzyme inhibitors; ARB: angiotensin receptor blocker; STEMI: ST elevation myocardial infarction

### Potential factors account for the regular-and-off-hour mortality difference

Table 3 showed that OR of death is 0.80 for patients in regular hour group compared to those in off-hour group, after adjusting for age, gender and education. Further adjustment for patients’ risk factors plus history of disease, disease severity, and in-hospital treatments could lead to a change by 5.0%, 55.0% and 20.0% in relative risk difference attributed to admission time, respectively. And all variables together resulted in a change of 70.0%, and the residual relative risk became no longer significant. Further analyses showed that for “non-severity” patients who were Killip < 3 without need for resuscitation, mortality in regular hour group were similar with that in off-hour group, 3.5% versus 3.9% respectively (P = 0.39).

**Table 3:** In-hospital mortality OR of admission time and proportion of relative risk difference attributed to admission time that could be explained by additional variables in different models.

Generalized linear mixed models	OR of admission time	Percent of effect of admission time accounted ^a^
Base model: Admission time + Age + Gender + Education	0.80 (0.67–0.95)	-
Model 1: Base model + Disease history and risk factors ^b^	0.81 (0.68–0.97)	5.0
Model 2: Base model + Disease severity ^c^	0.91 (0.74–1.11)	55.0
Model 3: Base model + All Treatments ^d^	0.84 (0.70–1.01)	20.0
Model 4: Base model + All above variables	0.94 (0.77–1.15)	70.0

^a^ Calculated by using formula: $\frac{\left|\left(1\text{-}{\text{OR}}_{\text{i}}\right)-\left(1\text{-}{\text{OR}}_{\text{b}}\right)\right|}{1\text{-}{\text{OR}}_{\text{b}}}\times 100\%$, Where OR_b_ represents OR of admission time from base mode and OR_i_ represents OR of admission time after adding explaining variables.

^b^ Disease history and risk factors include stroke and obesity.

^c^ Disease severity include systolic blood pressure<90mmHg, heart rate> = 100beats/min, Killip class, cardiopulmonary resuscitation and abnormal rhythm of heart within 24 hours of admission.

^d^ All treatments include thrombolytic therapy, aspirin and angiotensin converting enzyme inhibitors / angiotensin receptor blocker within 24 hours of admission.

## Discussion

Like the early studies in United States and other countries [2], the present study of STEMI patients in China demonstrated that the in-hospital mortality was significantly higher for patients admitted during off-hour than regular hour. However, the regular-and-off-hour disparity of in-hospital mortality was relatively smaller in our study patients, comparing with that reported in Isogai’s study [13]. The recent emphasis on timely treatment through early use of evidence-based therapies such as percutaneous coronary intervention, thrombolytic therapy and medications for STEMI patients and establishing “green channels” that patients with chest pain have priority to receive medical checks, and diagnosed STEMI patients could receive reperfusion therapy and medications immediately in emergency department may be the reason [14–16]. And in United States, studies have shown that the regular-and-off-hour difference in mortality had been diminished recently after the gaps in treatments were closed [3]. In our study, we found that patients admitted during off-hour received less evidence-based medications such as aspirin, angiotensin converting enzyme inhibitors or angiotensin receptor blocker, beta-blocker and statin. However, the absolute differences between the groups were small at about 2% to 4%, and percent of receiving thrombolytic therapy was even higher in patients admitted during off-hour than those during regular hour. Multiple analyses showed that the medical treatments could only explain 20% of the relative risk difference attributed to admission time. It has to be noted that still only 35–43% of STEMI patients received thrombolysis among these Chinese resource-constrained hospitals. This is too low considering that the timely PCI is not available in these hospitals and thrombolytic therapy should be recommended as an alternative.

Unlike previous studies, we found that majority of the excess death in patients admitted during off-hour could be attributed to the difference in disease severity at admission, much larger than that attributed to medical treatments [7]. In our study, patients admitted during off-hour had a higher proportion of hypotension, tachycardia, Killip IV, cardiopulmonary resuscitation and abnormal rhythm of heart within 24 hours of admission, in comparison with those admitted during regular hour. And disease severity could attribute to as much as 55% of the excess death in the off-hour group. Although we are the first to reveal that the excess death in off-hour patients with STEMI may be attributed to disease severity, the higher severity of the disease in off-hour patients had been reported in other studies. AMIS Plus Registry study and a nationwide study in Japan both showed a higher proportion of Killip III/IV and shock in patients admitted during off-hour [13,17]. There were also reports that patients with disease onset at midnight had longer ischemic time and consequently larger myocardial infarction size [18–19]. But it still remains unclear why STEMI patients admitted during off-hour presented with a higher severity of disease. Since longer pre-hospital delay was observed in regular hour group in our study, we may suspect that patients with minor myocardial infarction size onset at off-hour such as mid-nights might tend to wait till next regular hour for medical care, but further studies are required to clarify this question. The results indicated that public education to increase the awareness of STEMI early signs and the importance of timely treatment should be promoted as the next major action to reduce the mortality from STEMI. After many years of promotion, the timely initiation of reperfusion therapy and evidence-based medications among STEMI patients may have reached the peak, and the rooms for further improvement have become quite small. For example, over 95% and 90% of patients received aspirin and statin within 24 hours in our study. However, the average time from onset to arriving hospital was 4 hours, indicating that most patients were not able to receive thrombolytic therapy within 3 hours. To further improve the STEMI care, measures to shorten the hours from onset to admission must be developed and put into action.

To our knowledge, this is the first report to reveal the mortality disparity of admission time in real-world patients with STEMI in China, and also the first prospective study with large sample size to quantitatively evaluate factors that could account for this mortality disparity.

Meanwhile, the interpretation of our results required caution due to the nature of observational study that we cannot account of unmeasured and unknown confounders. However, observational cohort study is the only option since randomized controlled trail is not feasible to address such question. And we did not collect information on biomarkers, cardiac imaging, etc. Their roles in explaining the regular-and-off-hour disparity could not be analyzed or adjusted in our analyses. Another limitation was lack of data regarding medication contraindications and there is possibility that less medications in off-hour group may be due to more contraindications. In addition, our study only included level two hospitals in China and might limit the extrapolation of our conclusion.

## Conclusions

This large observational registry-based study demonstrated that STEMI patients admitted during off-hour in Chinese level two hospitals had higher in-hospital mortality than those admitted during regular hour. This higher mortality was attributed primarily to disease severity at admission and secondly to the poorer medical treatments. The results indicated that public education to increase the awareness of STEMI early signs and the importance of timely treatment should be promoted as next major action to reduce the mortality from STEMI.

## Supporting information

S1 Table Detail definitions of main variables. (DOCX)
