# Supplementary material for: Factors attributed to the higher in-hospital mortality of ST elevation myocardial infarction patients admitted during off-hour in comparison with those during regular hour
Source: PLoS One. 2017 Apr 7;12(4):e0175485. doi: 10.1371/journal.pone.0175485 (PMC5384766; doi:10.1371/journal.pone.0175485)
Supplement: S1 Table — (DOCX) [file pone.0175485.s001.docx]

S1 Table. Detail definitions of main variables

| Variables | Definition |
| --- | --- |
| Disease severity at admission |  |
| Hypotension | Systolic blood pressure<90mmHg measured at first present to emergency room or hospital. |
| Tachycardia | Heart rate>=100beats/min measured at first present to emergency room or hospital. |
| Killip class | Killip class is made based on functional capacity:  Killip class I includes individuals with no clinical signs of heart failure;  Killip class II includes individuals with rales in the lungs, an S3 gallop, and elevated jugular venous pressure;  Killip class III includes individuals with frank pulmonary edema;  Killip class IV includes individuals in cardiogenic shock. |
| Cardiopulmonary resuscitation within 24h | Receiving cardiopulmonary resuscitation within 24 hour of admission. |
| Abnormal rhythm of heart within 24h | Ventricular tachycardia, ventricular fibrillation, or atrial fibrillation was detected from continuous electrocardiogram monitoring within 24 hour of admission. |
| Complications |  |
| Heart failure | There are signs or symptoms in compliance with heart failure, combined with congestion documented by chest X ray, or diuretics commenced. |
| Major arrhythmia | Persistent ventricular tachycardia or ventricular fibrillation or Mobitz type II or III atrio-ventricular block happened in hospital. |
| Bleeding | Intracranial haemorrhage or a >5 g/dL decrease in haemoglobin concentration or a >15% absolute decrease in haematocrit. |
| Reoccurred myocardial infarction | Acute myocardial infarction was defined in accordance with the European Society of Cardiology/American College of Cardiology consensus definition. For patient admitted as acute myocardial infarction, if the following criteria are satisfied, the patient is considered as having in hospital re-infarction:   \| Clinical scenario \| Criteria \| \| --- \| --- \| \| Recurrent symptoms occurring within 18 hours \| Chest pain lasting >=30 minutes and >=2 mm of ST elevation \| \| Recurrent symptoms occurring after lasting 18 hours \| CK rise to >2×ULRR and>50% above previous baseline value  or CK-MB value>ULRR and>50% above previous baseline value or new left bundle branch block or new Q waves \| |
| Stroke | There are signs or symptoms concordant with stroke happened in hospital, and the diagnosis was verified by Computed tomography or Magnetic resonance imaging. |
| Primary outcome |  |
| In-hospital death | Died in hospital. |

CK: creative kinase; CK-MB: creative kinase MB; ULRR: upper limit of the reference range.
